# Amifostine protects from the peripheral sensory neuropathy induced by oxaliplatin in mice

**DOI:** 10.1590/1414-431X202010263

**Published:** 2020-09-18

**Authors:** A.F. Pereira, J.A. Lino, B.W.F. Alves, M.R.P. Lisboa, R.B. Pontes, C.A.V.G. Leite, R.B. Nogueira, R.C.P. Lima-Júnior, M.L. Vale

**Affiliations:** 1 Departamento de Fisiologia e Farmacologia, Faculdade de Medicina, Universidade Federal do Ceará, Fortaleza, CE, Brasil; 2 Departamento de Medicina Clínica, Faculdade de Medicina, Universidade Federal do Ceará, Fortaleza, CE, Brasil; 3 Departamento de Morfologia, Faculdade de Medicina, Universidade Federal do Ceará, Fortaleza, CE, Brasil; 4 Departamento de Fisioterapia, Faculdade de Medicina, Universidade Federal do Ceará, Fortaleza, CE, Brasil

**Keywords:** Oxaliplatin, Amifostine, Neuropathy, Chemotherapy, Pain

## Abstract

Sensory neuropathy is a dose-limiting side effect of oxaliplatin-based cancer treatment. This study investigated the antinociceptive effect of amifostine and its potential neuroprotective mechanisms on the oxaliplatin-related peripheral sensory neuropathy in mice. Oxaliplatin (1 mg/kg) was injected intravenously in Swiss albino male mice twice a week (total of nine injections), while amifostine (1, 5, 25, 50, and 100 mg/kg) was administered subcutaneously 30 min before oxaliplatin. Mechanical and thermal nociceptive tests were performed once a week for 49 days. Additionally, c-Fos, nitrotyrosine, and activating transcription factor 3 (ATF3) immunoexpressions were assessed in the dorsal root ganglia. In all doses, amifostine prevented the development of mechanical hyperalgesia and thermal allodynia induced by oxaliplatin (P<0.05). Amifostine at the dose of 25 mg/kg provided the best protection (P<0.05). Moreover, amifostine protected against neuronal hyperactivation, nitrosative stress, and neuronal damage in the dorsal root ganglia, detected by the reduced expression of c-Fos, nitrotyrosine, and ATF3 (P<0.05 *vs* the oxaliplatin-treated group). In conclusion, amifostine reduced the nociception induced by oxaliplatin in mice, suggesting the possible use of amifostine for the management of oxaliplatin-induced peripheral sensory neuropathy.

## Introduction

Oxaliplatin is a third-generation platinum compound commonly used for the treatment of metastatic colon cancer (1). Despite significantly increasing patients' survival (2,3), oxaliplatin causes several side effects that reduce their quality of life. The main side effect is neurotoxicity, manifested as peripheral sensory neuropathy (4) accompanied by significant neuronal damage in sensory nerves (5). Additionally, long-term treatment may affect proprioception, causing motor impairment (1). Symptoms of neuropathy, such as tingling, numbness in hands and feet, and pain, are common in patients treated with oxaliplatin, which may persist for more than five years (6). Hospitalization and dose reduction are common consequences of these side effects (7), which also cause treatment discontinuation (8). The management of peripheral sensory neuropathy symptoms includes antidepressants, anticonvulsants, opioids, and local anesthetics, among other drugs (9). However, there is no fully effective drug in preventing or reducing these symptoms, or even therapy capable of modifying the development of such toxicity.

Amifostine is a broad-spectrum cytoprotective agent that selectively protects non-tumor tissues from the toxic effects (10), such as nephrotoxicity (11) and neurotoxicity (12), induced by several chemotherapeutic regimens (13). Amifostine demonstrates a potent analgesic and anti-inflammatory activity in experimental models of inflammation (14). However, there is limited and controversial clinical evidence of the effects of this drug on platinum compounds-related neuropathy (15,16).

Therefore, this study investigated the antinociceptive effect of amifostine and the possible neuroprotective mechanism on the experimental peripheral sensory neuropathy induced by oxaliplatin in mice.

## Material and Methods

### Animals

The experiments were performed using Swiss albino male mice (*Mus musculus*), weighing 25-30 g, obtained from the Animal Facility of the Federal University of Ceará (Brazil). The animals were randomly divided into seven experimental groups (n=6) and accommodated in appropriate cages with controlled temperature (22±2°C) and light/dark cycle (12/12 h), receiving solid food and water *ad libitum*. The experiments were approved by the Ethics Committee in Animal Research of the Federal University of Ceará (protocol 27/08) and followed the local guidelines on the welfare of experimental animals.

### Induction of the peripheral sensory neuropathy

According to Azevedo et al. (17), oxaliplatin (Sigma-Aldrich^®^, USA), diluted in a 5% glucose solution (Dinâmica Química Contemporânea Ltda., Brazil), was administrated intravenously (1 mg/kg, twice a week, with a total of nine injections) in the lateral vein of the mice's tail. In the vehicle control group, a 5% glucose solution was administered intravenously. Mechanical and thermal nociceptive tests were conducted up to the 49th experimental day to evaluate the long-term effects of the oxaliplatin injections.

### Evaluation of the protective effect of amifostine on the oxaliplatin-induced peripheral sensory neuropathy

Amifostine (Sigma-Aldrich^®^) was dissolved in sterile saline solution and administered at the doses of 1, 5, 25, 50, or 100 mg/kg, twice a week (total of nine injections) by the subcutaneous route 30 min before the injection of oxaliplatin (18). The oxaliplatin-treated group received the same volume of sterile saline solution. Mechanical and thermal nociceptive tests were performed to evaluate the protective effect of amifostine up to the 49th experimental day.

### Behavioral tests

#### Plantar mechanical hyperalgesia test

The electronic von Frey apparatus (Insight^®^, Brazil) was used to evaluate the intensity of plantar mechanical hyperalgesia of each animal. This equipment records the pressure in grams required to provoke a hind paw flexion followed by a flinch, through a mechanical stimulus of the paw by the tip of a rigid filament. The animals were housed in acrylic boxes (12×10×17 cm) under a wire grid 10 min before the behavioral test. A trained researcher, blind to the treatments, evaluated the mechanical hyperalgesia once a week before and after the drug injections (17,19).

#### Cold allodynia tail immersion test

The tail immersion test was performed to evaluate the cold allodynia induced by a non-noxious temperature of 10°C (20,21). The total duration of the tail immersion was recorded in seconds, weekly, with a cut-off time of 120 s by a blind experimenter. One week before the experiment, the animals were adapted to the test.

#### Immunofluorescence

On the 28th experimental day, the dorsal root ganglia (DRG) were harvested for immunofluorescence assay. The mice were deeply anesthetized with an intraperitoneal administration of ketamine (100 mg/kg) (König do Brasil Ltda, Brazil) and xylazine (10 mg/kg) (König do Brasil Ltda). The anesthesia procedure was required considering the intracardiac perfusion that preceded the euthanasia method used in this study for the fixation of internal organs. The animals were intracardially perfused using 40 mL of sterile saline followed by 40 mL of a 4% paraformaldehyde (PFA) (Sigma-Aldrich^®^) solution. The DRG were then immersed in 4% PFA for 2 h followed by cryoprotection with a 30% sucrose solution for two days. After this, the tissues were inserted in the Tissue-Tek O.C.T. compound (Sakura^®^, The Netherlands) and stored at a temperature of -80°C. Serial sections of the frozen DRG samples were acquired (10-μm thick) with a cryostat (Leica CM1850, Leica, Germany).

For the immunofluorescence assay, sample sections were fixed in methanol (Vetec Química Fina Ltda, Brazil), followed by antigenic recovery in 0.1 M (pH 6.0) citrate buffer, at a temperature of 95°C. To allow the permeabilization of the nuclear membrane, 0.1% Triton X-100 (Sigma-Aldrich^®^) was used. Then, a solution containing 5% bovine serum albumin (Sigma-Aldrich^®^) added to 0.3 M glycine (Sigma-Aldrich^®^) was used to block the non-specific antibody binding. The incubation of the primary antibodies was carried out overnight with rabbit anti-c-Fos (Santa Cruz Biotechnology^®^, USA), in a 1:200 dilution, rabbit anti-nitrotyrosine (Merck Millipore^®^, USA), in a 1:300 dilution, and rabbit anti-activating transcription factor 3 (ATF3) (Abcam^®^, UK), in a 1:300 dilution. After this, the sections were incubated with the secondary antibody goat anti-rabbit IgG coupled to Alexa Fluor 568 (Invitrogen^®^, Life Technologies, Thermo Fisher Scientific, USA), at a dilution of 1:400. The NeuN antibody conjugated with Alexa Fluor 488 (Merck Millipore^®^) was used to label the neuronal cell bodies, at a dilution of 1:150. Slides were mounted with ProLong Gold Antifade Mountant (Invitrogen^®^, Life Technologies, Thermo Fisher Scientific). The slides were photographed using a laser scanning confocal microscope (Zeiss LSM710, Carl Zeiss, Germany). The photographs were carefully taken with the same master gain and digital offset for all the samples, providing standardization for posterior analysis.

With the photomicrographs, the fluorescent area was quantified using an image analysis software (Fiji ImageJ, National Institutes of Health, USA). This quantification was blindly performed through the differentiation of the fluorescent pixels by the higher color saturation associated with the fluorescence (green or red). The color threshold previously set the higher and lower limits to define the selected and unselected pixels. The results obtained with the quantification of the fluorescent area were calculated by the positive fluorescent area concerning the fluorescence of NeuN and are reported in percentage.

### Statistical analysis

The data are reported as means±SE. The statistical differences among the experimental groups in the behavioral tests were assessed by two-way analysis of variance (ANOVA) followed by Tukey’s post-test. To evaluate the other results, one-way ANOVA followed by Tukey’s post-test was used. The level of significance was considered to be P<0.05. GraphPad Prism version 6.00 for Windows (GraphPad Software, USA) was used for the analyses.

## Results

### Effect of amifostine on the mechanical nociceptive threshold in mice submitted to oxaliplatin-induced peripheral sensory neuropathy

**Figure 1:**
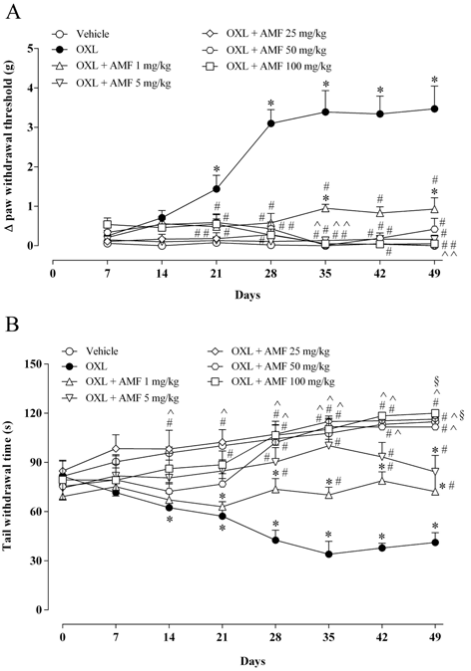
Effect of amifostine on behavioral tests. Oxaliplatin (OXL) (1 mg/kg) or 5% glucose (vehicle group) were injected intravenously, twice a week, for five weeks (a total of nine injections). Amifostine (AMF) (1, 5, 25, 50, or 100 mg/kg) was administered subcutaneously 30 min before the oxaliplatin injection. Von Frey electronic test (mechanical hyperalgesia) (**A**) and tail immersion test in cold water (10°C) (cold allodynia) (**B**) were performed once a week, for 49 days. The results are reported as means±SE. *P<0.05 *vs* vehicle; ^#^P<0.05 *vs* OXL; ^^^P<0.05 *vs* OXL + AMF 1 mg/kg; ^§^P<0.05 *vs* OXL + AMF 5 mg/kg (two-way ANOVA followed by Tukey post-test).

The injection of oxaliplatin increased the variation of the paw withdrawal threshold significantly from the 21st to the 49th day (P<0.05) compared with the vehicle group (Figure 1A). Subcutaneous administration of amifostine, at doses of 1, 5, 25, 50, and 100 mg/kg, prevented this increase of the variation from the 21st day until the 49th day (P<0.05), compared to the group treated with oxaliplatin (Figure 1A).

### Effect of amifostine on cold allodynia in mice submitted to oxaliplatin-induced peripheral sensory neuropathy

Oxaliplatin significantly reduced the tail withdrawal time from the 14th to the 49th day (P<0.05) compared with the group that received only the vehicle (Figure 1B). The subcutaneous injection of amifostine at doses of 1, 5, and 50 mg/kg prevented the decrease of tail withdrawal time from the 28th day until the 49th day (P<0.05), while the doses of 100 mg/kg prevented this from the 21st to the 49th day (P<0.05), compared to the oxaliplatin group (Figure 1B). Only the dose of 25 mg/kg was able to prevent this reduction from the 14th day until the 49th day (P<0.05) compared with the oxaliplatin group (Figure 1B). Thus, based on the behavioral results and total area under the curve (data not shown), the dose of 25 mg/kg was used for further analyses.

### Effect of amifostine on c-Fos expression in dorsal root ganglia

**Figure 2:**
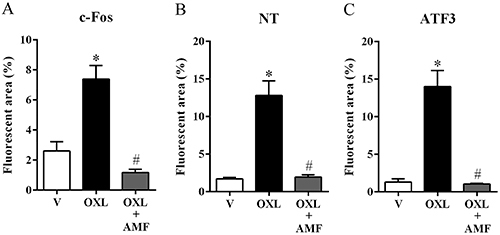
Fluorescent area quantification of c-Fos, nitrotyrosine (NT), and activating transcription factor 3 (ATF3) expression in the dorsal root ganglia of mice. The data are reported as means±SE of the percentage of positive fluorescent areas of c-Fos (**A**), NT (**B**), and ATF3 (**C**) expression concerning NeuN (neuronal marker) expression on the 28th experimental day. *P<0.05 *vs* vehicle group, ^#^P<0.05 *vs* OXL group (one-way ANOVA followed by Tukey post-test). AMF: amifostine; OXL: oxaliplatin; V: vehicle.

**Figure 3:**
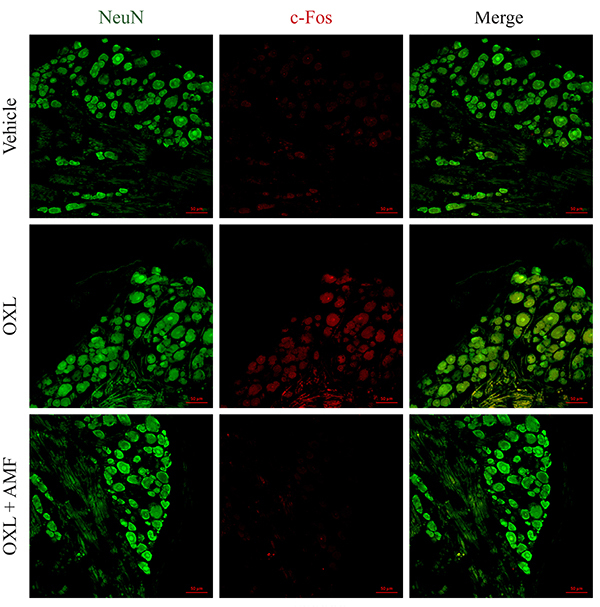
c-Fos expression in the dorsal root ganglia on the 28^th^ experimental day. Green: neuronal marker (NeuN); red: c-Fos; merge: yellow. Magnification 200×; scale bar 50 μm. OXL: oxaliplatin; AMF: amifostine.

The c-Fos immunofluorescence analysis is commonly used to demonstrate increased neuronal activity related to nociceptive behavior (22). Our results showed that oxaliplatin significantly increased c-Fos expression (P<0.05) in neuronal cells (labeled with NeuN) from the DRG of the mice compared with the vehicle group. Amifostine (25 mg/kg) treatment prevented the c-Fos increase (P<0.05) in neuronal cells compared to the oxaliplatin group (Figure 2A and Figure 3).

### Effect of amifostine on nitrotyrosine expression in dorsal root ganglia

**Figure 4:**
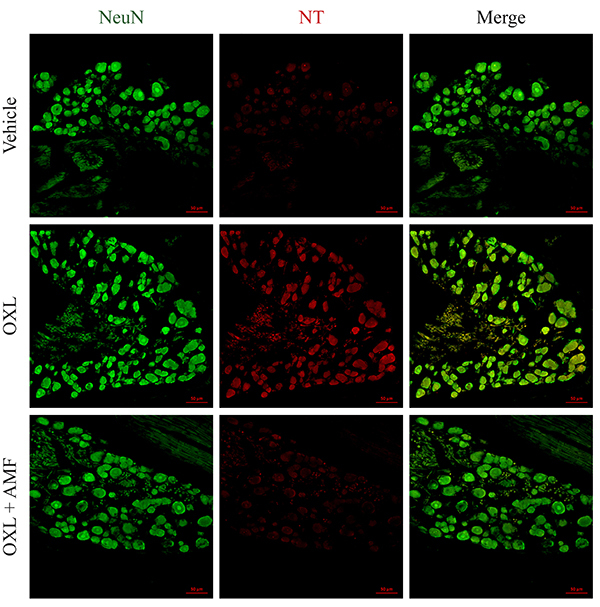
Nitrotyrosine (NT) expression in the dorsal root ganglia on the 28th experimental day. Green: neuronal marker (NeuN); red: nitrotyrosine; merge: yellow. Magnification 200×, scale bar 50 μm. OXL: oxaliplatin; AMF: amifostine.

Nitrotyrosine immunoexpression was evaluated to investigate a possible anti-nitrosative mechanism involving the amifostine neuroprotective effect. In the oxaliplatin-treated group, there was a significant increase of nitrotyrosine expression (P<0.05) in neuronal cells (labeled with NeuN) of the DRG compared to the vehicle group. The treatment with amifostine prevented the increase of the nitrotyrosine expression (P<0.05) in neuronal cells induced by the oxaliplatin injections compared with the oxaliplatin-treated group (Figure 2B and Figure 4).

### Effect of amifostine on ATF3 expression in dorsal root ganglia

**Figure 5:**
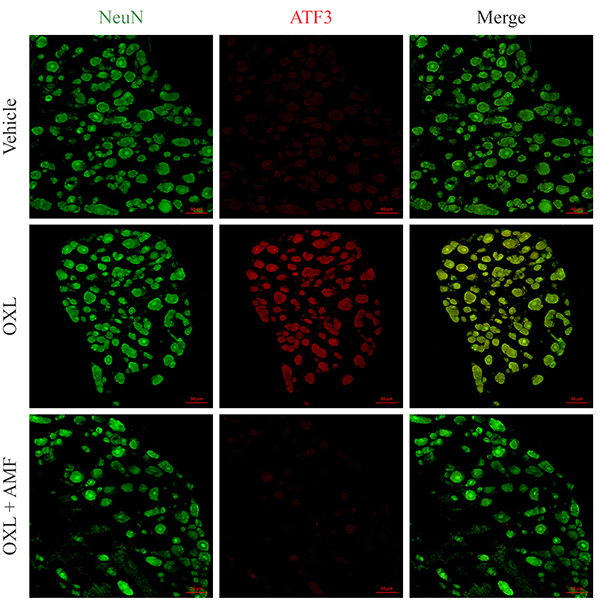
Activating transcription factor 3 (ATF3) expression in the dorsal root ganglia on the 28th experimental day. Green: neuronal marker (NeuN); red: ATF3; merge: yellow. Magnification 200×, scale bar 50 μm. OXL: oxaliplatin; AMF: amifostine.

ATF3 is a transcription factor expressed by neuronal cells and a known marker of neuronal injury and repair (23,24). In the oxaliplatin group, a considerable increase in the ATF3 expression (P<0.05) was found in neuronal cells (labeled with NeuN) of the DRG compared with the vehicle. Amifostine prevented this increase (P<0.05) in neuronal cells, compared to the oxaliplatin group (Figure 2C and Figure 5).

## Discussion

In the present study, the protective effect of amifostine was demonstrated in a model of peripheral sensory neuropathy induced by oxaliplatin in mice. This protection was confirmed by the modulation of neuropathy markers, such as c-Fos, nitrotyrosine, and ATF3.

Nociceptive behavioral tests were employed to confirm the development of neuropathy (17,21). As expected, oxaliplatin induced a nociceptive response detected by the mechanical and thermal nociceptive tests. Several mechanisms, including oxidative (26) and nitrosative stresses (17,27), contribute to the development of neuropathic pain after treatment with chemotherapeutic agents (25). In that context, drugs that target reactive oxygen and nitrogen species, such as amifostine, are of clinical interest. Amifostine, an inorganic thiophosphate, presents a cytoprotective effect of broad-spectrum selective to healthy tissues (10). The active metabolite of amifostine (WR-1065) can eliminate free radicals produced in response to chemical substances and ionizing radiation. This metabolite prevents platinum-DNA adducts formed by platinum compounds and promotes the decrease of DNA cross-links caused by NH_2_ groups of alkylating agents (10). The absence of the alkaline phosphatase enzyme in the tumor possibly explains the selective cytoprotective effect of amifostine on non-tumor cells. This enzyme is required to transform amifostine in its active metabolite. Then, amifostine does not interfere with the antitumor effect of antineoplastic drugs (10).

The neuroprotective effect of amifostine has been demonstrated in the neurotoxicity induced by platinum agents in some clinical trials (12,28). This effect was also confirmed in an *in vitro* model of oxaliplatin-induced neurotoxicity by using PC12 pheochromocytoma cells (29). Furthermore, amifostine shows a protective effect against 5-fluorouracil-induced hyposalivation, possibly by preventing inflammation (30) and neuronal damage.

Considering that the metabolites of amifostine do not accumulate in the central nervous system due to the blood-brain barrier (10), we decided to study the effect of amifostine on the peripheral nervous system. Oxaliplatin increased the c-Fos expression in neuronal cells in the DRG, as previously demonstrated (21). This increase of c-Fos expression was also shown in the dorsal horn of the spinal cord (17) and neural tissues following the oxaliplatin injection (31,32). The increased c-Fos expression suggests neuronal activation and neuroplasticity (22,33). Basal levels of c-Fos are usually low, but afferent stimuli during painful conditions may induce its expression (22,34). Interestingly, amifostine prevented c-Fos immunoexpression and neuronal activation caused by the treatment with oxaliplatin, suggesting an inhibitory effect upon nociceptive stimulation.

Azevedo et al. (17) showed that oxaliplatin increases nitrotyrosine expression in the neuronal cells of the spinal cord dorsal horn. In the present study, the increase of nitrotyrosine expression was also found in peripheral nervous system neuronal cells. Nitrotyrosine is formed through the reaction of peroxynitrite (ONOO^-^) with the residues of tyrosine amino acids, and is a marker of nitrosative stress (35), potentially contributing to mitochondrial dysfunction (36). In the central and peripheral nervous systems, reactive oxygen species mediate the neurotoxicity consequent to the use of chemotherapeutic agents, such as oxaliplatin (37,38). In this study, we showed that amifostine prevented the increase of nitrotyrosine expression in the DRG of mice treated with oxaliplatin, suggesting the inhibition of nitrosative stress. The preventive effect of amifostine against nitrosative stress was previously demonstrated in a liver damage model induced by gamma irradiation in rats (39). Other compounds with antioxidant properties, such as the flavonoids rutin and quercetin, have been effective in preventing the nociceptive symptoms and the observed neuronal changes in oxaliplatin-neurotoxicity (17). Thus, antioxidants agents, such as amifostine, are promising compounds for preventing the peripheral sensory neuropathy induced by oxaliplatin (17,21).

Given that amifostine prevented neuronal hyperactivation and nitrosative stress, its effect on the expression of ATF3 was further investigated. ATF3, a marker of neuronal injury, is an activating transcription factor/cyclic AMP response element-binding protein (ATF/CREB) family member expressed by several tissues during stress conditions (23). In the present study, oxaliplatin increased ATF3 expression in neuronal cells of the DRG. The DRG neuronal damage was also shown by Di Cesare Mannelli et al. (40). Moreover, Pereira et al. (21) showed that ATF3 expression remains increased after interruption of oxaliplatin administration, suggesting non-reversible damage. In basal conditions, ATF3 expression is not observed in the DRG and spinal cord neurons (23). Conversely, the expression of this marker increases in neurons submitted to axotomy. Furthermore, it has been shown that ATF3 exerts an essential role in nerve regeneration by increasing the intrinsic growth of injured neurons (24). Notably, amifostine prevented the increase of ATF3 expression in the DRG neurons, showing its protective effect against the neuronal damage caused by oxaliplatin.

In conclusion, amifostine demonstrated a neuroprotective effect on oxaliplatin-induced neurotoxicity. It was confirmed by the prevention of nitrosative stress, neuronal hyperactivation, and damage. This work supports the use of amifostine as a potential strategy for preventing the development of peripheral sensory neuropathy induced by oxaliplatin.
